# The Practitioners' Bookshelf

**Published:** 1893-03-04

**Authors:** 


### THE PRACTITIONERS' BOOKSHELF.

### "THE CHEMISTRY OF LIFE AND HEALTH."*

This is a short illustrated University Extension Series manual dealing with the sanitation of life from the chemical standpoint. After a short chapter on the general principles of chemistry, the author goes on to show how those principles are applied in the performance of the functions of life, pointing out as he goes along the mischief arising from the wrong application of them, and how necessary a knowledge of them is to every one. In short, it is an excellent, not too popularly treated physiological chemistry. The language is easy, plain to be understood, and the explanation sufficient. It is just the book that an educated adult, wishing to know something of the reason of his being and of the surroundings which keep that being alive and in health, should read. There is a good index and a number of useful illustrations. We congratulate Dr. Kimmins on its production, and hope he will add to the series yet others as good.

* "The Chemistry of Life and Health." By C. W. Kimmins, M.A., D.Sc., Staff Lecturer in Chemistry Cambridge University Extension Scheme. (Methuen and Co., 1892, 167 pp., Illustrated.)

### PROGRESS OF HYGIENE.*

We have received from the Medical Library of O. Berthier a catalogue of publications dealing with medical hygiene in the Argentine Republic, and a report on the progress of public hygiene in that republic by Dr. Emile Coni, presented at the International Congress held in London in 1891.

The subjects dealt with in the report are infantile hygiene, including the care of exposed children, school hygiene, adulteration of food, industries, naval and military hygiene, water supply, drains, maintenance of the town, public lavatories, cremation (which we are glad to see has taken a firm hold of the inhabitants), public offices for vaccination and ambulances, &c., epidemic diseases, lock hospitals, inspection of imported animals, Pasteur's treatment for rabies, bacteriological laboratories, isolation hospitals, medical inspection of ships, and the Institution of Hygiene.

A short account of the creation of the regulations ruling these subjects is given, which is full of interest, as showing how a new country will often go ahead of the older ones.

* "Les Progrès de L'Hygiene Publique dans la République Argentine," par le Dr. Emile R. Coni. (Librairie Médicale de O. Berthier, Paris, 1891, p. 56.) "Catalogue des Publications Hygieniques et Médicales de la République Argentine." Ditto.

